# Barriers to HIV Testing Among Young Men Who Have Sex With Men (MSM): Experiences from Clark County, Nevada

**DOI:** 10.5539/gjhs.v8n7p9

**Published:** 2015-11-03

**Authors:** Jennifer R. Pharr, Nancy L. Lough, Echezona E. Ezeanolue

**Affiliations:** 1 School of Community Health Sciences, University of Nevada, Las Vegas, NV, USA; 2 College of Education, University of Nevada, Las Vegas, NV, USA

**Keywords:** HIV testing, barriers, men who have sex with men, young people

## Abstract

Clark County, Nevada had a 52% increase in newly diagnosed HIV infections in young people age 13-24 with 83% of the new diagnoses in this age group being men who have sex with men (MSM). HIV testing and counseling is critical for HIV prevention, care and treatment, yet young people are the least likely to seek HIV testing. The purpose of this study was to identify barriers and facilitators to HIV testing experienced by young MSM in Clark County, Nevada. We conducted a qualitative focus group discussion to identify barriers and facilitators to HIV testing among eleven young MSM in March, 2015. The primary barrier to HIV testing identified by the group was a lack of awareness or knowledge about testing for HIV. Other barriers within the person included: fear of results, fear of rejection, and fear of disclosure. Barriers identified within the environment included: access issues, stigma, and unfriendly test environments for young people. In addition to increasing awareness, intervention to increase HIV testing among MSM young people should incorporate access to testing in environments where the adolescents are comfortable and which reduces stigma. HIV testing sites should be convenient, accessible and young person/gay friendly.

## 1. Introduction

The World Health Organization (WHO), UNICEF and United Nations Population Fund (UNPFA) define young people as persons age 10-24 (UNDESA, N.D.). This group is especially vulnerable to HIV with young people aged 15-24 representing 39% of new HIV infections globally in people over the age of 15 (UNAIDS, 2014). In 2013, young people 13 to 24 years old accounted for 21% of new HIV infections in the United States (Hyden, Allegrante, & Cohall, 2014). One third of HIV positive Americans are young people between the ages of 13 and 29 (amfAR, 2010).

The incidence of new HIV infections is of particular concern in Nevada which ranked 26^th^ in the number of new HIV diagnoses in 2011 (Centers for Disease Control and Prevention, 2013). Clark County is home to nearly seventy-five percent of the population of Nevada and between 2009 and 2013, had approximately 350 new HIV diagnoses per year (Nevada Division of Public and Behavioral Health, 2014). New diagnoses of HIV infections among young people age 13-24 increased 52% from 2009 to 2013. In 2013, 83% of new HIV diagnoses among this age group were among men who have sex with men (MSM) (Nevada Division of Public and Behavioral Health, 2014).

HIV testing and counseling is critical for HIV prevention, care and treatment (Kurth, Lally, Choko, Inwani, & Fortenberry, 2015). The Centers for Disease Control and Prevention (CDC) recommends routine screening for HIV infection for all patients aged 13-64 years (CDC, 2007). Yet, the proportion of young people in the US who have received HIV testing has remained low and unchanged since 2005, at 22% of their population (Kann et al., 2014). Sixty-five percent of people 18 to 24 years old report they have never been tested, making them the least likely age demographic to seek HIV testing and the most likely to be unaware of their HIV status (CDC, 2014).

Several barriers to HIV testing have been identified. Young people rate confidentiality as the most important component of sex related health services (Committee on Pediatric AIDS, Emmanuel, & Martinez, 2011; Hyden et al., 2014). Peralta and colleagues (2007) found the most common reason for not being tested for HIV among 12-24 year olds was simply that they had never been offered a test. Concerns regarding cost and inability to easily access testing were also reported as common barriers to testing in young people in the United States (Peralta et al., 2007). Among young people globally, lack of confidentiality was the greatest barrier to testing with subjects also reporting fear of partner rejection, societal rejection, and job loss (Bhoobun et al., 2014). Fear of stigmatization and perception of HIV as extremely fatal were reported as common barriers to testing among young people in Africa (Bhoobun et al., 2014).

The majority of young people that have sex do not believe they are at risk for contracting HIV (Committee on Pediatric AIDS et al., 2011; Peralta et al., 2007). Fifty-four percent of young people surveyed by Peralta and colleagues stated they did not believe they could be HIV positive because they ‘don’t feel sick’ or ‘don’t feel that they were at risk for having HIV’, which indicates inadequate knowledge regarding the nature of early HIV infection, and may also be a barrier to testing in young people (Peralta et al., 2007).

Barriers to HIV testing have also been found among MSM. Major barriers to testing included gay-related stigma and HIV-related stigma, fear of disclosure (e.g., sexual orientation not known to parents), relationship type, partner characteristics, low perception of their risk of HIV, fear that HIV is incurable or being positive equals death, concern of confidentiality, unaware that testing may be offered for free, communication barriers with health professionals and name-based testing (Dowson, Kober, Perry, Fischer, & Richardson, 2012; Schwarcz et al., 2011; Wei et al., 2014; Wong et al., 2012). Song and colleagues found that barriers to HIV testing among young MSM living in China included: a perceived low risk of HIV infection, fear that others will find out about their orientation, not wanting to know their HIV status, not knowing where to go for testing and inconvenience of testing (Song et al., 2011).

Despite the low rate of HIV testing among young people and the increase in newly HIV diagnosed young MSM, not much is known about barriers to HIV testing among adolescent MSM in the United States. In this study, we utilized Bandura’s Social Cognitive Theory (SCT) to identify barriers or facilitators to HIV testing that reside within the individual-knowledge, self-efficacy, goals and outcome expectations, as well as barriers or facilitators that reside within the social or structural environment (Bandura, 1994, 2004). The purpose of this study was to identify barriers as well as facilitators to HIV testing experienced by young MSM in Clark County, Nevada to inform future interventions to increase testing among this group.

## 2. Methods

### 2.1 Study Design

We conducted a qualitative focus group discussion to identify perceived barriers and facilitators to HIV testing among young MSM in March, 2015. In afocus group, a facilitator leads a discussion on a specific topic and the group typically includes six to twelve people (Green & Thorogood, 2009). Focus groups are particularly effective in providing information about why people think or feel the way they do rather than focusing on gathering uniform information on representative or random samples (Krueger, 2009). The focus group was semi-structured which allowed for a more in-depth discussion around answers to the scripted questions (Dowson et al., 2012; Green & Thorogood, 2009)

### 2.2 Setting and Population

Purposive sampling was used to recruit participants age 18 to 24. Participants were recruited by the staff at the Lesbian, Gay, Bisexual, Transgender, and Queer (LGBTQ) Center (The Center) in Las Vegas, NV. The focus group discussion was also conducted at The Center. A total of eleven young people participated in the semi-structured focus group discussion.

### 2.3 Data Collection

Upon arrival, participants were told the purpose of the study and asked to read and sign a consent form. The discussion was led by one member of the research team with expertise in qualitative research and transcribed by two other members of the team. The discussion lasted for ninety minutes. Questions included: What are things that you have experienced or that you know other young people have experienced that make it difficult to get HIV testing (barriers)? What are things that you have experienced or that you know other young people have experienced that make it easier to have HIV testing (facilitators)? What would you do to reduce barriers and/or increase facilitators to HIV testing for young people? We included the phrase “or that you know other young people have experienced” so that participants felt safe to talk about experiences they might have had but feel uncomfortable talking about by attaching the experience to another person. The discussion was conducted in a relaxed manner which allowed time for everyone to speak and share their thoughts and the facilitator encouraged participation which was well received by the group (Green & Thorogood, 2009).

### 2.4 Data Analysis

We used the SCT to code barriers and facilitators to HIV testing that reside: 1) within the person (i.e. knowledge, fear, self-esteem) or 2) within the person’s environment (i.e. cost of testing, unfriendly test environment) (Bandura, 2004). Thematic content analysis involved review of transcripts to extract themes (Green & Thorogood, 2009). Two researchers from the team analyzed the focus group transcripts. Themes were identified and then coded as either residing within the person or within the person’s environment. Quotations were grouped according to the themes. Throughout the data analysis, themes were always verified by two of our experienced qualitative researchers. They coded transcripts separately to encompass a wider analytical net and achieved a base agreement percentage of 90%. This is one of Osborne’s four strategies for assessing the validity of qualitative data (Osborne, 1990).

Quotations were assigned pseudo names to provide anonymity for the participants. This study was approved by the University of Nevada, Reno Institutional Review Board.

## 3. Results

Of the eleven participants in the focus group, two were transgender (1 male-to-female and 1 female-to-male), one was bisexual and eight were gay men (Table 1). Five were White, four were Hispanic and two were multi-racial (one Asian-Hispanic and one Black-Asian). The age range was 18-24 with a mean age of 22.3.

**Table 1:** Demographic Characteristics of the Participants

Race/ethnicity	N	%
White	5	45.5
Hispanic	4	36.3
Asian/Hispanic	1	0.1
Black/Asian	1	0.1

Age		

20	1	0.1
21	3	27.3
22	3	27.3
23	0	0.0
24	4	36.3

Orientation/identity		

Gay	8	72.7
Bisexual	1	0.1
Transgender	2	18.2

### 3.1 Factors Within the Person

Several barriers to HIV testing were found that resided within a person (Table 2). Themes included: lack of awareness or knowledge, fear, and lack of self-esteem or confidence. All of the participants identified a lack of awareness or knowledge about testing for sexually transmitted diseases (STDs) in general and HIV specifically as a barrier to testing. The participants discussed that they knew diseases could be spread through sex and that abstinence and condom use could prevent the spread of diseases, but that they could not remember being told that they should be tested for STDs and HIV if they were sexually active. One participant stated:

**Table 2:** Barriers and Facilitators to HIV Testing Within the Person and Within the Structural Environment

Barriers	Facilitators
**Within the Person**	**Within the Person**

*Lack of awareness or knowledge*	
*Fear of:*	*Fear* about having HIV can result in testing
•results/fear of being HIV positive	
•rejection from family/community	
•disclosure to family	
*Lack of self-esteem or confidence*	

**Within the Environment**	**Within the Environment**

*Access*	*Access*
•Cost of testing	•Ability to call information and get testing locations
•Lack of transportation	•Ads in local gay publication that list testing sites and cost of testing
•Lack of phone	•Online information about testing site from the public health department
•Wait time for results (blood tests)	•Mobile testing
•Concerns about parental consent for under 18	•Availability of rapid HIV testing
	
*Stigma*	
•Stigma that HIV is a gay disease	
•Lack of support from the community for gay people	
*Unfriendly test environments*	*Friendly test environments*
•Uncomfortable environment created by some health professionals (specifically doctors)	•Comfortable environment with patient friendly counselors (The Center)
•Crowded testing sites	

I was just completely unaware about being tested for HIV until I came to The Center and saw flyers for free HIV testing. I just never thought about being tested until I saw the flyers. (Mike)

Another participant added:

In sex ed in schools, they talked to us about puberty and sexually transmitted diseases, how to prevent them. We learned how to put a condom on a banana and stuff like that, but they never talked about getting tested for STDs. (Sam)

One participant had attended a magnet high school for students interested in health professions. He said that they were taught about transmission of STDs and HIV from a medical perspective, but information about testing such as when or where a person should get tested was not covered. The participants discussed at length that they thought the lack of comprehensive sexual education in the Clark County School District (CCSD) was the cause of the lack of knowledge about the importance of testing for STDs because testing was not discussed at school. Eight of the eleven participants had attended high school in CCSD.

Fear of being HIV positive, rejection, and disclosure were also identified as barriers; however, fear of being HIV positive was considered to be both a barrier and a facilitator to HIV testing. One participant stated that:

Fear is a big factor that keeps people away. Young people are paralyzed by fear. (Chris)

But participants also talked about how being afraid of having HIV can motivate some people to get tested.

Fear of rejection and disclosure were also discussed as barriers to HIV testing among gay young people. One participant talked about wanting to be tested but still being “in the closet”. He said:

I was afraid that if my parents found out that I had been tested for HIV that they would also find out that I was gay and I was not ready to come out to my parents. (James)

The participants discussed how they perceive that HIV is still considered to be a gay disease. If someone is positive then it is assumed that they are gay. The participants felt there was not support from the community for gay people and that disclosure could result in rejection from family and the community.

The last barrier to HIV testing that resides within a person discussed by participants was lack of self-esteem or self-confidence. They identified this as a barrier to HIV testing as well as seeking information about HIV.

### 3.2 Factors Within the Environment

Several barriers and facilitators to HIV testing were identified within the environment (Table 2). Themes included: access (lack of transportation, lack of phone, cost of test, concerns about parental consent for patients under 18 years old, wait time for results), stigma (HIV is still seen as a gay disease, lack of support from the community for gay/homosexual people) and unfriendly test environments for young people (uncomfortable environment created by some health professionals, crowded testing sites). One of the participants talked about getting ‘the look’ [of disapproval] from his doctor when he asked for a referral for HIV testing. Other participants confirmed having similar experiences when interacting with some medical professionals. However, they also discussed how friendly and compassionate medical professions make it easier to get testing, and they had all experienced supportive medical professionals as well. Facilitators to testing included the ability to get information about HIV testing such as calling information, finding information on the internet and in local gay publications. Additionally, the participants discussed how some settings make it easier from them to be tested including access to mobile and rapid testing and young person/gay friendly environment such as that at The Center.

### 3.3 Recommendations to Improve HIV Testing Rates

The participants made recommendations to improve HIV testing rates among young people in the community. The majority of the recommendations focused on education and making testing readily available at high schools, where young people spend most of their time. Suggestions included: adding HIV and STD testing information to the sex education curriculum, talking about HIV and STD testing during school assemblies, and distributing flyers and pamphlets about the importance of HIV testing and testing locations. Additionally, the participants suggested sexual health services be incorporated into the Health Services currently provided on school campuses. Recommended sexual health services included STD and HIV testing, pregnancy testing and condom distribution.

## 4. Discussion

Many behavior theorists posit that a person must have knowledge about a disease and disease prevention in order to engage in prevention behaviors (Albarracín et al., 2005; Albarracín, Durantini, & Earl, 2006; Bandura, 1994, 1998, 2000, 1977; Fishbein, 2000; Fishbein & Yzer, 2003; Glanz, Rimer, & Viswanath, 2008). Low levels of HIV knowledge and lack of HIV education are associated with reduced use of HIV services (Schunter, Cheng, Kendall, & Marais, 2014). The participants identified lack of awareness and knowledge about getting testing for HIV or any STD as the greatest barrier to HIV testing in the community. Not surprisingly, their recommendations to improve HIV testing focused on educating young people about the importance of testing including why, when and where to get tested. CCSD is the 5^th^ largest school district in the US (American Schools and Universities, 2014). The sexual education curriculum in CCSD is not comprehensive and is abstinence based (CCSD, 2014a, 2014b). Important information remains excluded from the curriculum including: resources for screening and treatment for sexually transmitted diseases and other related communicable diseases, resources for effective and safe methods of contraception and contraceptive devices, and information on gender stereotypes (CCSD, 2014b). Additionally, there is no requirement that the methods of teaching and the materials used by teachers ensure nondiscriminatory instruction for all pupils, regardless of their race, gender, gender identity or expression, religion, sexual orientation, ethnic or cultural background or disability (CCSD, 2014b).

The content of sexual education in high school varies greatly in the US; however, studies have shown that abstinence-based sexual education is less effective than comprehensive education for HIV prevention (Hall, Moreau, & Trussell, 2012; Lindberg, Santelli, & Singh, 2006; Lindberg & Maddow-Zimet, 2012; Ott & Santelli, 2007; Santelli, 2008; Vivancos, Abubakar, Phillips-Howard, & Hunter, 2013). The lack of information about screening and treatment for STDs and safe methods of contraception along with no training for teachers about how to present sexual education in a nondiscriminatory way puts young people in Clark County at increased risk for STDs, teen pregnancy and can lead to stigmatization of LGBTQ people. Both formal (comprehensive sex education in school) and informal (social media, social marketing) education could be used to increase knowledge about HIV testing among this population (Bleakley, Hennessy, Fishbein, & Jordan, 2009; Futterman et al., 2001; Jones & Biddlecom, 2011; Lagus, Bernat, Bearinger, Resnick, & Eisenberg, 2011; Secor-Turner, Sieving, Eisenberg, & Skay, 2011).

Similar to findings from Song and colleagues, the focus group talked about their fears of rejection and disclosure as well as HIV-related and homosexuality-related stigmas in the community (Song et al., 2011). Globally, MSM and transgender young people face issues with HIV testing as they are often hidden and it is not safe for them to reveal their sexual identity to health professionals or family (Kurth et al., 2015). The perception of stigma, of both homosexuality and HIV, from the wider culture has been found to be a major barrier to HIV testing among MSM globally and in our study (Lorenc et al., 2011). This issue seems to transcend culture and resources and is found in high-income settings such as the US as well as resource limited settings such as Africa (Kurth et al., 2015; Song et al., 2011). Continued efforts to de-stigmatize HIV and homosexuality both globally and locally are needed and could start with health professionals. Many health professionals in the US do not discuss sexual orientation with their sexually active young people because they perceive that they lack the skills to do so. A start to creating a safe space for young MSM and transgender young people could be to provide health professionals with skills for discussing sexual orientation with their patients (Kitts, 2010).

Several barriers and facilitators to HIV testing in the environment were identified by the focus group. Accessibility of testing as well as young person friendly and LBGTQ friendly testing sites are important for uptake of HIV testing among this population (Kurth et al., 2015). It is important for them to be able to find information about HIV testing costs and locations easily (i.e. the internet, local publications), for locations to be convenient (i.e. mobile testing) and testing to be timely (i.e. not crowded and with rapid testing available) and for the health professionals to be friendly and supportive. One barrier that the participants identified which was truly a perceived barrier rather than an actual barrier was parental consent. As of 2015, Nevada as well as 49 other states allows minors to consent to have STD testing and treatment. Eleven of the states have a minimum age requirement which varies between twelve and fourteen years of age. Thirty-one states explicitly include HIV testing and treatment in the STD services available to minors. No states require physicians to inform parents that their minor has requested testing or treatment with the exception of one state (Iowa) that requires physicians to notify a minor’s parents if the HIV test is positive (Guttmacher Institute, 2015). This again points to the importance of educating young people about the availability of and their rights to access STD and HIV testing and treatment.

## 4.1 Limitations

We recognize that there are limitations to this analysis. As with any research, especially qualitative, there is a potential for research bias. We attempted to minimize this bias by having two skilled qualitative researchers code the data. A limitation of qualitative research is the inability to generalize the results; however, the findings do point to some important directions for more broad based studies and can guide the development of interventions for this group.

## 5. Conclusion

HIV testing is a critical step for HIV prevention and treatment. The high rates of HIV infection and low rates of HIV testing among young people, particularly MSM and transgender youth, is concerning. Interventions to increase HIV testing among this group should include HIV testing sites that are convenient, accessible and young person/gay friendly in addition to an education component which addresses the importance of HIV testing (why, when and where). Community efforts are needed to reduce the stigma of HIV and homosexuality.
